# Germline *BRCA1* Mutation Detected in a Multiple Endocrine Neoplasia Type 2 Case With *RET* Codon 634 Mutation

**DOI:** 10.3389/fgene.2019.00544

**Published:** 2019-06-11

**Authors:** Balázs Sarkadi, Kornélia Baghy, Zoltán Sápi, Gábor Nyirő, István Likó, Attila Patócs

**Affiliations:** ^1^HAS-SE “Lendulet” Hereditary Endocrine Tumors Research Group, Hungarian Academy of Sciences, Semmelweis University, Budapest, Hungary; ^2^2nd Department of Medicine, Semmelweis University, Budapest, Hungary; ^3^National Bionics Program, Budapest, Hungary; ^4^1st Department of Pathology and Experimental Cancer Research, Semmelweis University, Budapest, Hungary; ^5^HAS-SE Molecular Medicine Research Group, Hungarian Academy of Sciences, Semmelweis University, Budapest, Hungary; ^6^Department of Laboratory Medicine, Semmelweis University, Budapest, Hungary

**Keywords:** medullary thyroid cancer, *RET* mutation, *BRCA1* mutation, multiple endocrine neoplasia type 2, cancer genetics

## Abstract

Coincidences of more than one pathogenic mutation in high and/or moderate risk-associated cancer genes have been rarely reported, and the implication for disease progression has been debated. We present a case harboring two autosomal dominant inherited mutations potentially aggravating the phenotype.

**Case report:** A 16-year-old female was referred to the Endocrine Unit due to two palpable thyroid nodules and hair loss. Two hypoechoic, inhomogeneous masses with microcalcification in the thyroid gland were confirmed as medullary thyroid carcinoma. Genetic testing revealed a pathogenic heterozygous *RET* mutation associated with multiple endocrine neoplasia type 2 (MEN2). Furthermore, genetic screening identified the same mutation in the proband’s clinically negative brother as well as in his two sons. The proband’s mother and maternal aunt died of breast cancer. No samples were available from the deceased. The proband underwent further genetic counseling and *BRCA1/2* testing. A novel, frameshift heterozygous *BRCA1* mutation (*BRCA1* p.Ile90Serfs, NC_000017.10:g.41256905_41256917) was identified in the proband, but it was absent in the brother and father, indicative of maternal inheritance. Breast or ovarian cancer was neither detected in our case at initial presentation nor during the 6-year follow-up.

**Conclusion:** Coincidence of two monogenic autosomal dominant tumor syndromes is extremely rare, but it represents a significant therapeutic and cancer surveillance challenge. Due to the wider use of next generation sequencing in clinical practice, similar situations may occur more frequently.

## Background

Hereditary cancers account for 5–10% of all cancers. The identification of these patients and their presymptomatic genetically affected family members is critical because of the necessity of specialized diagnostic and therapeutic approaches. Of classical autosomal inherited tumor syndromes, multiple endocrine neoplasia type 2 (MEN2), with the underlying mutations of the *RET* proto-oncogene, is one of the best characterized diseases. Patients with MEN2 syndrome are disposed to develop medullary thyroid carcinoma (MTC), pheochromocytoma (PHEO), and hyperparathyroidism (HPT). As a result of large international efforts, clear genotype-phenotype associations have been reported (Mathew et al., 1987; Machens et al., 2005), and codon-specific genetic counselling can be offered for affected patients (Milos et al., 2008). However, the penetrance of manifestations varies even in the same family (Milos et al., 2008). This variability in phenotype could be linked to many factors including other genetic alterations (Whitworth et al., 2016, 2018; Stradella et al., 2018), genetic modifiers (Lendvai et al., 2012), environmental factors, and epigenetic events.

Germline inactivating variants in the tumor suppressor genes *BRCA*1 and *BRCA*2 confer high lifetime risks of breast cancer as much as 72 and 69%, respectively; ovarian cancer as much as 44 and 17%, respectively; and less frequently also other cancers (Kuchenbaecker et al., 2017). *BRCA1* has a role in DNA repair, transcriptional response to DNA damage and DNA damage-responsive cell cycle regulation (Yoshida and Miki, 2004).

Here, we report a 16-year-old female patient with MEN2*-*associated MTC caused by the heterozygous mutation of *RET* codon 634, as well as a pathogenic, heterozygous frameshift *BRCA1* mutation. To the best of our knowledge, this is the first report of a patient with both pathogenic *RET* and *BRCA1* mutation.

## Materials and Methods

All genetic analysis was performed within the frame of the routine clinical genetic testing service at Clinical Genetics and Endocrinology Laboratory Semmelweis University following the institutional protocol. Genomic DNA was extracted from peripheral blood leukocytes using the DNA isolation kit for mammalian blood (Boehringer Mannheim Corporation, Indianapolis, IN, USA), and mutation analysis of *RET* and *BRCA1/2* genes was carried out using polymerase chain reaction (PCR) followed by direct bidirectional Sanger sequencing of the PCR-amplified DNA using BigDye^®^ Terminator v3.1 Cycle Sequencing Kit’ and ABI PRISM^®^ 3100 Genetic Analyzer (Thermo Fischer Scientific, Waltham, MA, USA). The study was conducted in accordance with the Declaration of Helsinki and has been approved by the Scientific and Research Committee of the Medical Research Council of Hungary (ETT-TUKEB 4457/2012/EKU). Written informed consent for publication in behalf of the proband was obtained from the parent since the proband was 16 years old at the time of the diagnosis. All subjects gave written informed consent in accordance with the Declaration of Helsinki.

## Case Report

A 16-year-old female patient was investigated for hypothyroidism due to hair loss. During the routine physical examination, two thyroid nodules were identified; therefore, the patient was referred to the Endocrine Unit of the 2nd Department of Medicine, Semmelweis University, for further endocrinological investigation in 2013. Thyroid Stimulating Hormone (TSH) level was within the physiological range (2.44 mU/L; reference value: 0.35–4.94 mU/L). Due to the present thyroid nodules thyroid ultrasound examination was carried out. Two hypoechoic, inhomogeneous masses with microcalcification were identified in both lobes of the thyroid gland. Biopsy and fine-needle aspiration biopsy (FNAB) were performed. Pathological examination revealed invasive growth of tumor cells with coarsely granulated chromatin and high number of mitosis. Calcitonin immunohistochemistry showed strong positivity in 100% of the cancer cells. Computerized tomography (CT) confirmed two masses: in the right lobe the tumor diameter was 2.2 cm, whereas in the left lobe 0.6 cm. The patient’s serum calcitonin (501 pg/ml; reference value: 0–6 pg/ml) and carcinoembryonic antigen (CEA, 7.4 ng/ml; reference value: 0–4.3 ng/ml) levels were markedly elevated. Plasma parathormone level (37.9 pg/ml; reference value: 15–65 pg/ml) and 24-h collected urine metanephrine (178 mcg/24 h; reference value: 64–302 mcg/24 h) and normetanephrine (385 mcg/24 h; reference value: 162–527 mcg/24 h) levels were within reference range.

A total thyroidectomy was performed and pathological examination of the specimen confirmed MTC. Metastases were detected in three lymph nodes removed from the left side, whereas four lymph nodes removed from the right side were metastasis free and no distant metastasis was detected. The TNM classification of the tumor was pT2N1a (Wells et al., 2015). Although the serum calcitonin and CEA levels significantly decreased after the surgery, complete biochemical remission has not been achieved since, and the serum calcitonin levels has shown uncertain progression in the range of 31–178 ng/L during the clinical follow-up in the last 6 years. Based on the postoperative staging CT scan, one small residual thyroid tissue was suspected without any sign of metastases. Repeated ultrasound and FNAB confirmed the lesion to be scar tissue and did not affirm the presence of residual thyroid tissue. Thyroid stimulating hormone levels increased after the total thyroidectomy to 31.1 mU/L, and after successful thyroid hormone substitution with L-thyroxine, physiological values were reached as TSH concentrations varied between 0.128 and 3.843 mU/L (reference value: 0.35–4.94 mU/L).

**Figure 1:**
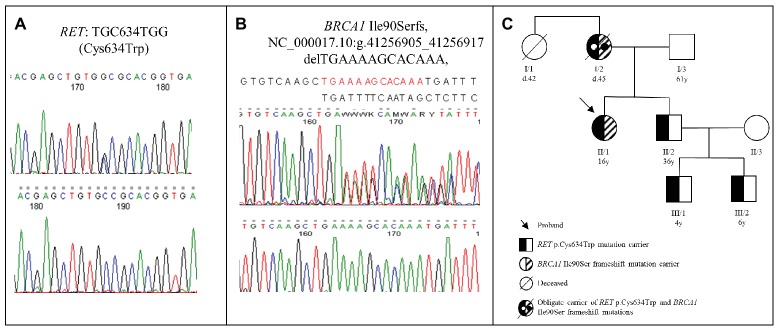
**(A)** Chromatogram of the *RET* Cys634Trp mutation identified in the proband. **(B)** Chromatogram of the frame shift *BRCA1* Ile90Ser mutation identified in the proband. **(C)** Pedigree of the proband’s family. The proband (II/1) was 16 years old at the time of the diagnosis (currently 22 years old), while his brother (II/2) was 36 years old (currently 42 years old). The proband’s brother’s sons were 4 years old (III/1) (currently 10 years old) and 6 years old (III/2) (currently 12 years old) at the time of the genetic counselling. The proband’s mother (I/2) was 34 years old at the time of the breast cancer diagnosis and died at the age of 45 due to brain metastases. The proband’s aunt (I/1) was 36 years old at the time of the breast cancer diagnosis and died at the age of 42 (d, age at death; y, years).

After genetic counseling, the patient underwent genetic testing (Wells et al., 2015) of *RET* gene, and a pathogenic heterozygous mutation (p.Cys634Trp) was identified (Figure 1A). Two other synonymous frequent RET polymorphisms (rs1800860 and rs1800861) were also detected. The p.Cys634Trp is a well-known mutation associated with MEN2A syndrome; therefore, the patient underwent detailed clinical, laboratory, and imaging studies for manifestations of MEN2A. Adrenal and/or extra-adrenal paraganglial tumors were ruled out by CT imaging of the head, neck, chest, and abdominal region. No other neoplastic lesions were observed. Biochemical data for free catecholamines and metanephrines in the 24 hour urine revealed no sign of catecholamine or metanephrine secreting tumor. Serum intact parathormone was within the normal range, and no sign of parathyroid malfunction was detected. During the last 6-year follow-up, the proband is still free of any MEN2A associated lesions.

Due to the fact that MEN2A syndrome is inherited in an autosomal dominant way, genetic counseling and genetic testing of first degree relatives are indicated (Wells et al., 2015). The detailed family history revealed that the patient’s mother and her aunt had breast cancer and both died due to brain metastases. This unexpected clinical presentation was suspicious to hereditary breast cancer syndrome. However, no samples for mutation analysis of *BRCA1*/2 genes were available from these two affected relatives. Hereditary breast cancer is also an autosomal dominant disorder mutation, and thus, analysis is recommended for the first-degree relatives (Moyer, 2014). Accordingly, the mutation analysis of *BRCA1*/2 genes identified a pathogenic, heterozygous frameshift *BRCA1* mutation (*BRCA1* Ile90Serfs, NC_000017.10:g.41256905_41256917 delTGAAAAGCACAAA) in the proband (Figure 1B). Clinical examination and breast magnetic resonance imaging (MRI) revealed no sign of malignancy in the breasts, and 6 years after initial examination still no signs of breast or ovarian cancer are present. After these striking results, the patient’s family members underwent genetic counseling again and, after obtaining informed consent, underwent mutation testing for the *RET* exon 11 and *BRCA1* genes. The proband’s brother (age: 36 years) and his two sons (age: 4 and 6 years) were also heterozygotes for the *RET* p.C634W mutation, but the *BRCA1* mutation was absent in them. In her brother, two thyroid masses and elevated serum calcitonin level (215 pg/ml) were observed. Total thyroidectomy was performed, and his serum calcitonin returned to normal range. Two sons of the proband’s brother underwent prophylactic thyroidectomy, and the histopathological examination of the removed tissues showed no signs of MTC. The proband’s father did not carry the *RET* mutation and the *BRCA1* mutation. The pedigree of the family is shown in Figure 1C.

## Discussion

To the best of our knowledge, we present the first patient who carries both *RET* and *BRCA1* pathogenic germline mutations. The combination of two hereditary cancer syndromes is extremely rare and represents a challenging situation regarding routine follow-up screening and patient’s management. The prevalence of MEN2 syndrome is approximately 1:30,000 (Kloos et al., 2009), whereas the estimated prevalence of *BRCA1/2* mutations in general population varies between 1:330 and 1:500 (Moyer, 2014). Therefore, co-harboring a *RET* and *BRCA1* mutations is extremely rare; it may be approximately 1:10–15,000,000. This is in line with data deposited in a database containing cases where two pathogenic mutations in genes encoding hereditary cancers were identified. In this database, only 39 cases have been uploaded (Stradella et al., 2018). It should be noted that in NGS cancer gene panel *RET* or *MEN1* genes are not studied. A panel sequencing of 64 genes by Shin et al. revealed that of 252 breast cancer patients, 67 patients (26.8%) carried 77 pathogenic mutations (12 in *BRCA1*, 13 in *BRCA2*, 9 in *CDH1*, 3 in *FH*, 5 in *MSH2*, 2 in *MSH6*, 4 in *NAT1*, 6 in *PTCH1*, 3 in *RAD51*, 7 in *RET*, 4 in *SPINK1*, 3 in *TP53* and one each in *ALK*, *BRIP1*, *CHEK2*, *MLH2*, *MUTYH*, and *PTEN*). However, all these seven *RET* mutations were identified in *BRCA* negative cases (Shin et al., 2017).

Based on the pedigree and genetic analysis (absence of the *RET* mutation in our proband’s father), it is very likely that our proband’s mother was an obligate carrier of both RET p.Cys634Trp and BRCA1 Ile90Ser frameshift mutations. The phenotype associated with this particular *RET* mutation is well summarized (Milos et al., 2008). Based on an international database, the death cause for this mutation was due to PHEO in 50% and metastatic MTC in 19% of cases. Until now, neither PHEO nor HPT was revealed in any of our *RET* mutation carriers by regular screening performed as recommended (Wells et al., 2015). However, our proband carries a pathogenic *BRCA1* mutation, which could be responsible for death of her mother and aunt. Our proband underwent a complex screening procedure according to the ESMO Clinical Practice Guideline, and at this time, no alterations in breasts and ovaries were detected. It should be mentioned that during regular follow-ups, a close screening and risk-reducing measures will have to be taken into account (Paluch-Shimon et al., 2016). Based on a recent clinical study, a more severe phenotype is not expected in this patient compared to those which would be developed if the mutations were alone (Stradella et al., 2018). However, from tumor biological point of view, it is unknown whether the coincidence of the *RET* and *BRCA1* mutations might aggravate clinical manifestations. Both the ovary and the breast express RET protein (Uhlen et al., 2015), and BRCA1 is also expressed in the thyroid gland (Uhlen et al., 2015). Several other tyrosine kinase receptor-related signal transduction pathways and tumor suppressors, including p53, are involved in tumorigenesis and progression of MTC (Santarpia et al., 2009). The tumor suppressor p53 can arrest the cell cycle in case of DNA damage in order to allow DNA to be repaired or can induce apoptosis if the DNA repair was unsuccessful (Levine, 1997). The absence of normal p53 function could contribute to RET-induced MTC development (Santarpia et al., 2009). BRCA1 and p53 form stable complexes, and BRCA1 was found to be a potent coactivator of p53-dependent transcription of the p21 and *BAX* genes; therefore, BRCA1 is a critical cofactor of p53 in its tumor suppression role (Zhang et al., 1998). Based on clinical presentation, Li-Fraumeni syndrome caused by germline *TP53* mutation was not suspected, but as mutant p53 might have a role in our family, we sequenced the whole coding region of the *TP53* gene. No pathogenic mutation but a Pro72 variant in both the proband and her brother was detected (data not shown). Altogether, our results may suggest that the mutant *BRCA1* in our proband may be associated with a more aggressive MTC (it developed at younger age with lymph node metastasis) compared to her older brother without *BRCA1* mutation. However, we cannot exclude entirely that the *RET* C634W mutation contributed for this intra-familial difference because the penetrance of MEN2A-associated lesions for this particular *RET* mutation showed large variability in affected patients presented in a large international study (Milos et al., 2008).

From the therapeutic point of view, it is crucial to distinguish the metastatic lesion attributed to MTC from another coexisting primary malignancy. Extrathyroidal malignancies rarely accompany MTC such as breast or brain cancer along with other cancers (Alevizaki et al., 2013). Gagel et al. (1988) reported a patient with MEN 2A syndrome who died because of metastatic breast cancer during the clinical follow-up of thyroidectomy, which was carried out due to a metastatic MTC. Unfortunately, no other information was given about the patient regarding familial history of breast cancer or *BRCA1/2* mutation analysis.

In summary, we have presented the first reported case of a patient carrying pathogenic *RET* and *BRCA1* mutations. The recent next-generation sequencing-based technologies are providing more and more genetic data including incidental findings, but as our case showed even the association of various hereditary cancer syndromes may be expected. These patients will require multidisciplinary cooperation as well as individual approaches to achieve the best tumor surveillance and therapy.

## Ethics Statement

The study was conducted in accordance with the Declaration of Helsinki and has been approved by the Scientific and Research Committee of the Medical Research Council of Hungary (ETT-TUKEB 4457/2012/EKU). All subjects gave written informed consent in accordance with the Declaration of Helsinki.

## Author Contributions

AP conceived the study. BS and AP designed the research. ZS and KB histopathologically analyzed the patients’ samples. AP evaluated patients. GN and AP performed genetic screening. BS, IL, and AP analyzed the data. BS and AP wrote the manuscript.

### Conflict of Interest Statement

The authors declare that the research was conducted in the absence of any commercial or financial relationships that could be construed as a potential conflict of interest.
